# O10 A case of sarcoidosis mimicking Sjögren’s syndrome along with abnormal nailfold capillaroscopy

**DOI:** 10.1093/rap/rkaa053.009

**Published:** 2020-11-03

**Authors:** Ziad Alkutobi, Arslan Sidhu, Anupama Nandagudi

**Affiliations:** Basildon and Thurrock University Hospitals, Basildon, United Kingdom

## Abstract

**Case report - Introduction:**

Sicca symptoms and Raynaud’s disease are amongst the common presentation of Sjögren’s syndrome (SS) which has an estimated prevalence of 1%. We are reporting a case that was initially considered as SS, but subsequent lip biopsy confirmed histological evidence of sarcoidosis in the presence of abnormal nailfold capillaroscopy and negative autoimmune profile. Sarcoidosis can be associated with various autoimmune diseases including systemic sclerosis, Hashimoto's thyroiditis, systemic lupus erythematosus, rheumatoid arthritis, Sjögren’s syndrome and spondyloarthropathy.

**Case report - Case description:**

A 29-year-old Caucasian man was referred to rheumatology clinic, suffering with ongoing sicca symptoms including dry itchy gritty eyes, sore red eyelids, persistent mouth dryness and cracking ulcers. He has history of Raynaud’s disease, dysphagia for solid, dry cough and photosensitive rash on his chest and arms. His other symptoms were polyarthralgia, myalgia, headache, persistent fatigue, poor memory, dry skin over the hands and tingling sensation at the feet. There was no history of joint swelling, uveitis, chest pain or shortness of breath. He has hypothyroidism secondary to radioiodine treatment and is currently on levothyroxine. He works in a factory, drinks alcohol occasionally and is non-smoker. There was no family history of autoimmune conditions.

General and systemic examination was unremarkable, and there were no stigmata of connective tissue disease except for tenderness and crepitus at the right knee joint. His saliva flow rate was 0.4ml/5 mins and Schirmer’s test was 20mm/5 mins (both were normal values).

Autoimmune profile was negative; ANA: 0.2 units, RF factor: <10, CCP antibodies (IgG): 1.1U/mL.   Apart from mild lymphopenia, the other laboratory tests were at normal range including CK, CRP, adjusted calcium, ACE, immunoglobulins, serum protein electrophoresis and urine test for protein. Chest X-ray and lung function test were both normal.

Nailfold capillaroscopy shows grade1 enlarged capillaries, prominent capillary ramifications in the form of bushy capillaries and some microhaemorrhages, a pattern suggestive of mixed connective tissue disease.

Lip biopsy showed small non-caseating epithelioid granuloma within the parenchyma.

The initial working diagnosis was a possible serology-negative SS, but the later results of lip biopsy confirmed sarcoidosis.

He was commenced on Hydroxychloroquine and advised about cognitive-behavioural therapy for generalised ache and fatigue. As he was not responding to pilocarpine, a referral for ophthalmology review was requested.

**Case report - Discussion:**

Sarcoidosis is a multisystem disease of unknown aetiology, known to exist worldwide with a variable prevalence of around 20 per 100,000 in the UK. It is characterized by the presence of multiple non-caseating granulomas, inflammation which may occur in any tissue in the body manifested as local symptoms with or without systemic features. Involvement of the salivary and lacrimal glands can result in xerostomia and xerophthalmia, respectively.

Chronic sarcoid has an often subtle, insidious, progressive, and highly variable clinical course; it can be asymptomatic in a significant percentage of the cases.

In addition to the clinical and radiological findings, the diagnosis of sarcoidosis should be based on the histological proof of non-caseating granulomas and ruling out other diseases that shares similar features.

SS has many features in common with sarcoidosis; these may include sicca symptoms, arthralgia, myalgia, arthritis, erythematous rash, lymphadenopathy, peripheral neuropathy, fatigue and raised inflammatory markers. Moreover, positive rheumatoid factor, defective T suppressor cell regulation and HLA-DR3 are linked to both diseases. Pulmonary involvement in either of them may have similar clinical and radiographic manifestations, making it difficult for the clinician to distinguish between these diseases.

In terms of management, hydroxychloroquine may help the skin and joint manifestations in both diseases whereas more severe diseases may require treatment with glucocorticoids, methotrexate, and azathioprine.

Nevertheless, prompt confirmation of the diagnosis is crucial provided the difference in response to medication, complications, outcome, and prognosis.

Based on the clinical and lip biopsy findings, the above case was diagnosed with sarcoidosis. The case did not meet the criteria for the diagnosis of SS provided the negative results of Schirmer’s test, saliva flow test and autoimmune profile.

**Case report - Key learning points:**

Raynaud’s phenomenon (secondary Raynaud’s) with positive capillaroscopy findings was an unusual early presenting feature of sarcoidosis in the above case. There was one case report of similar presentation published in 2011. However, literature did not reveal specific information about abnormal nailfold capillaroscopy results in sarcoidosis.

Dry eyes and dry mouth may occur in sarcoidosis mimicking the presentation of primary SS. In addition, true coexistence of sarcoidosis and SS has also been defined based on the histopathologic examination of the exocrine glands. Diagnostic criteria need to be applied for patients with suspected overlap of the two diseases.

Clinicians will need to be vigilant and perform appropriate investigations for overlapping rheumatic conditions with sarcoidosis. Lip biopsy remains a crucial investigation for cases referred with sicca features, not only to establish the diagnosis of SS but also to exclude   associated lymphoma or sarcoidosis.

